# Psychometric properties of the NIH Toolbox Cognition Battery composites in older adults at risk for Alzheimer's disease and related dementias: A systematic review

**DOI:** 10.1002/alz.70673

**Published:** 2025-10-28

**Authors:** Amy B. Thomas, Allison L. Matthews, Olivia Brooks, Anna Winquist, Lonnie A. Nelson, Randi Arias‐Fontenot, Dedra Buchwald, Kaitlin B. Casaletto, Sandra Weintraub, Robert K. Heaton, Brian F. French, Astrid Suchy‐Dicey, Celestina Barbosa‐Leiker

**Affiliations:** ^1^ College of Nursing Washington State University Spokane Washington USA; ^2^ Department of Psychology Washington State University‐Tri Cities Richland Washington USA; ^3^ Washington State University Elson S. Floyd College of Medicine Spokane Washington USA; ^4^ College of Nursing University of Colorado Aurora Colorado USA; ^5^ Public Health Sciences Division Fred Hutchinson Cancer Center Seattle Washington USA; ^6^ Department of Neurological Surgery University of Washington Seattle Washington USA; ^7^ Memory and Aging Center, Department of Neurology, Weill Institute for Neurosciences University of California San Francisco California USA; ^8^ Mesulam Center for Cognitive Neurology and Alzheimer's Disease Northwestern University Feinberg School of Medicine Chicago Illinois USA; ^9^ Department of Psychiatry University of California San Diego La Jolla California USA; ^10^ College of Education Washington State University Pullman Washington USA; ^11^ Slone Epidemiology Center Boston University School of Medicine Boston Massachusetts USA; ^12^ College of Health and Human Development California State University Fullerton California USA

**Keywords:** Alzheimer's disease and related dementias, National Institutes of Health Toolbox Cognition Battery, psychometric properties, reliability, systematic review, validity

## Abstract

This systematic review evaluated the psychometric performance of the National Institutes of Health's Toolbox Cognition Battery (NIHTB‐CB) composite scores in older adults with and without Alzheimer's disease and related dementias (ADRD). A systematic literature search was conducted using MEDLINE, Embase, PsycINFO, and CINHAL databases. The evidence quality of NIHTB‐CB measurement properties was assessed using integrated Consensus‐based Standards for the Selection of Health Measurement Instrument (COSMIN) methodology and the Interpretation/Use Argument framework. Fourteen studies met inclusion criteria for this review of the NIHTB‐CB. Evidence supporting the scoring, generalization, and extrapolation inferences of the Total, Crystallized, and Fluid composite scores in older adults ranged from developing through exemplary ratings. Findings indicate additional research is warranted on the NIHTB‐CB in older adult and ADRD populations. The present study highlights the importance of continued use and research of the NIHTB‐CB in diverse, older populations who are at risk for ADRD.

**Highlights:**

- Limited research focuses on the National Institutes of Health's Toolbox Cognition Battery (NIHTB‐CB) composites in older adults.
- The general psychometric robustness of the NIHTB‐CB has been es.
- The Crystallized composite shows proficient psychometric evidence.
- The psychometric evidence of Fluid composite is developing due to limited data.

## 1 INTRODUCTION

Aging is the greatest known risk factor for Alzheimer's disease (AD) and related dementias (ADRD).^1^ At the group level, aging naturally coincides with the progression of subtle cognitive changes in processing speed and later progresses to gradual declines in executive function, memory, and visuospatial skills. Distinguishing between aging and disease‐related cognitive impairment, such as mild cognitive impairment (MCI), which is defined by disproportionate decline given one's age (and other person‐specific factors), can be challenging, especially in early stages of symptom development.^2^ Diagnosis of MCI and dementia relies on clinical assessment tools that often occur at a single time point and carry inherent limitations, which can contribute to misdiagnoses or can lead to misidentification of the people most at risk of disease burden. For example, unintentional test bias for marginalized individuals,^3^, ^4^, ^5^ systemic and interpersonal biases influencing diagnostic and referral practices,^6^, ^7^ and inconsistent cognitive test performance across different racial groups for cognitive tests used in current practice^8^, ^9^ can result in poorer outcomes in clinical care for non‐Hispanic Black groups compared to non‐Hispanic White groups.

Epidemiological data need to be accessible and inclusive to accurately reflect trends in prevalence and incidence of MCI and dementia. Due to the high and rapidly growing prevalence of ADRDs and the critical need for epidemiological data to accurately demonstrate the trends and factors influencing progression and diagnosis of ADRD, tools are needed that can identify and monitor cognitive decline in diverse older populations. The National Institutes of Health's Toolbox Cognition Battery (NIHTB‐CB) was designed as a neuropsychological assessment tool, comprising cognitive function measures that are useful in diverse research studies and across a wide age band (aged 3 to 85).^10^, ^11^ The main goal of the NIHTB initiative was to create a standard assessment tool for comparisons of cross‐study clinical research.^10^, ^12^ The NIHTB‐CB is widely used in community‐based and cross‐cultural epidemiological and clinical research^10^ and has been deployed in studies across multiple populations, including older populations with neurological impairments. Whether the NIHTB‐CB is an appropriate tool for distinguishing normal cognitive aging from MCI or dementia in older adults is yet unclear. To determine whether the performance of NIHTB‐CB is adequate for identifying ADRD and cognitive impairment in older populations, a systematic review was conducted to evaluate the psychometric properties of the NIHTB‐CB composite scores (i.e., Total, Fluid, and Crystallized) in older adults by applying the Consensus‐based Standards for the Selection of Health Measurement Instruments (COSMIN) methodology^13^, ^14^ integrated with the Interpretation/Use Argument (IUA) framework to build a validity argument.^15^, ^16^, ^17^

## 2 METHODS

### 2.1 Search strategy

A literature search was conducted using MEDLINE, Embase, PsycINFO, and CINHAL databases. The NIHTB debuted in 2012; thus, searches were limited from January 1, 2012, to June 20, 2024. The search terms used to identify the name of the instrument (NIHTB‐CB) were ([“National Institutes of Health Toolbox Cognition Battery” OR “NIH Toolbox Cognition Battery” OR “Toolbox Cognition Battery” OR “NIHTB‐CB”]). Terms to refine the searches and meet the objective of this study included ([“validity” OR “reliability” OR “repeatability” AND “dementia” AND “Alzheimer's disease”]). Additional parameters to refine the literature included: medical subject heading (MeSH) terms in MEDLINE (“psychometrics,” “cognition,” “reproducibility of results,” and “neuropsychological tests”), middle‐aged to older‐aged adult (45+ years) subjects, peer‐reviewed articles, and English‐language articles. Included references were also hand searched by the review team for any missed or additional articles for inclusion. This systematic review protocol is registered with PROSPERO (registration number: CRD42022359044).

#### 2.1.1 Criteria for study inclusion

Full‐text articles of studies on the measurement properties of the NIHTB‐CB were included. Eligible studies included participants who were self‐reported neurologically healthy older adults (≥ 49 years) and patients with dementia, MCI, traumatic brain injury (TBI), and stroke. We selected 49 years as our age cutoff to ensure our review was robust enough to capture individuals potentially at risk for early‐onset AD, which has a reported mean age of presentation of 56 years (± 5 years).^18^ Studies with TBI and stroke participants were included in the review because both diagnoses are major risk factors associated with developing ADRD.^19^, ^20^ Articles reporting measurement properties of the NIHTB‐CB in other patient populations were excluded to ensure the review focused on older adults and participants with ADRD‐related neurological impairments. Studies were not eligible if the mean age of the reported sample within the article was < 49 years. Articles were ineligible if data from older adults could not be extrapolated for review and analysis (e.g., pediatric data or younger adult data were analyzed without age strata) or if the study failed to contain applicable psychometric data on the measurement properties. Full‐text reviews of the studies with unclear psychometric evaluations were completed by two reviewers (A.B.T. and C.B.L.) independently and removed if the reviewers agreed that there was not a clear psychometric assessment. Studies that provided the Fluid, Crystallized, and/or Total scores for the sample—and the entirety of the Fluid and/or Crystallized tests to extract either the composite scores or the latent factor framework of the composite scores—were included in the review. Notably, there is much interest in the NIHTB‐CB to either validate novel measures or quantify cognitive abilities. Many articles were excluded by our review criteria because they reported NIHTB‐CB subtests individually but not composite scores. Arguably, the psychometric properties of the standalone subtests are relevant to the overall discussion of the performance of the NIHTB‐CB in older adults at risk for ADRD and should be assessed in the future; however, the scope of this systematic review was limited to the composite scores. More information on the NIHTB‐CB composite score inclusion criteria is provided in the section below.

#### 2.1.2 Composite scores of NIHTB‐CB

The NIHTB‐CB is a fully computerized, 30 minute cognitive screener available in English and Spanish. It assesses five core neurocognitive domains: executive functions, episodic memory, processing speed, working memory, and language. These domains are categorized into “Fluid” (encompassing the Dimensional Change Card Sort, Flanker Inhibitory Control and Attention, Picture Sequence Memory, List Sorting Working Memory, and Pattern Comparison Processing Speed tests) and “Crystallized” (assessed by the Picture Vocabulary and Oral Reading Recognition tests) abilities.^10^, ^12^ Weintraub et al.^21^ proposed gold‐standard neuropsychological examinations (Table 1) to assess the specific constructs targeted by the NIHTB‐CB. The NIHTB‐CB provides Fluid, Crystallized, and Total cognition (i.e., both Crystallized and Fluid) composite scores. Fluid neurocognitive abilities are represented as dynamic, biologically based, and sensitive to brain injuries or disease; Crystallized abilities are conceptualized as dependent on education and experiences (e.g., school, life experiences) and less affected by aging/disease. The NIHTB provides three score types for composites and subtests: unadjusted (i.e., compared to the full normative sample without demographic considerations), age adjusted (i.e., same age band), and fully adjusted (i.e., incorporating age, sex, race, ethnicity, and education). For a comprehensive understanding of these score types and their interpretation, readers are referred to the NIHTB‐CB Scoring and Inference Manual.^22^ Further information on the NIHTB‐CB can also be found in the foundational work by Gershon et al.^23^

1 **RESEARCH IN CONTEXT**

1. **Systematic review**: This systematic review examined how the National Institutes of Health Toolbox Cognition Battery (NIHTB‐CB) composite scores have been used in research involving older adults, both with and without Alzheimer's disease and related dementias (ADRD). We analyzed 14 relevant studies published up to June 20, 2024, identified through a comprehensive search of major research databases (MEDLINE, Embase, PsycINFO, and CINHAL).
2. **Interpretation**: The Total composite score was infrequently reported in the literature. Exemplary or proficient evidence supported the scoring, generalization, and extrapolation inferences of the Crystallized composite score. While evidence supported the use of generalization inference, only a limited number of studies provided developing evidence for scoring and extrapolation inferences in older adults with and without ADRD.
3. **Future directions**: Our synthesis of the literature demonstrates the general psychometric robustness of the NIHTB‐CB. Findings also indicate additional research is warranted on the NIHTB‐CB—specifically in older adult and ADRD populations. Future studies should focus on diverse groups of older adults at risk for ADRD to strengthen our understanding of how these cognitive measures perform.

**TABLE 1 alz70673-tbl-0001:** NIHTB‐CB subtests and corresponding convergent validation (“gold standard”) measures.^a^

Composite	Cognitive subdomain	NIHTB‐CB measure	“Gold standard” measure
Crystallized	Language, receptive	Picture Vocabulary	PPVT‐IV
	Language, expressive	Oral Reading Recognition tests	WRAT‐4
Fluid	Processing speed	Pattern Comparison Processing Speed	WAIS‐IV: Coding and Symbol Search
	Executive function, inhibitory control	Flanker Inhibitory Control and Attention	D‐KEFS: Color‐Word Interference
	Executive function, cognitive flexibility	Dimensional Change Card Sort	WCST‐64
	Episodic memory	Picture Sequence Memory	BVMT‐R, RAVLT
	Working memory	List Sorting Working Memory	WAIS‐IV: Letter‐Number Sequencing, PASAT

Abbreviations: BVMT‐R, Brief Visuospatial Memory Test—Revised; D‐KEFS, Delis–Kaplan Executive Function Scales; NIHTB‐CB, National Institutes of Health Toolbox Cognition Battery; PASAT, Paced Auditory Serial Addition Test; PPVT‐IV, Peabody Picture Vocabulary Test 4th Edition; RAVLT, Rey Auditory Verbal Learning Test; WAIS‐IV, Wechsler Adult Scale of Intelligence, 4th Edition; WCST‐64, Wisconsin Card Sorting Test‐64 Card version; WRAT‐4, Wide Range Achievement Test 4th Edition.

^a^ See Weintraub et al.^21^ for more information

To ensure a systematic comparison of the measurement properties for Fluid and Crystallized abilities across studies, articles were reviewed for both administration and scoring of the NIHTB‐CB within the studies. Articles that did not use the standard Fluid, Crystallized, or Total composite scoring, or the latent factor framework of the composites, were excluded from the review. All full‐text articles were reviewed by two reviewers (C.B.L. and A.L.M.) independently before being excluded.[Table alz70673-tbl-0001]

#### 2.1.3 Selection of included articles

**FIGURE 1:**
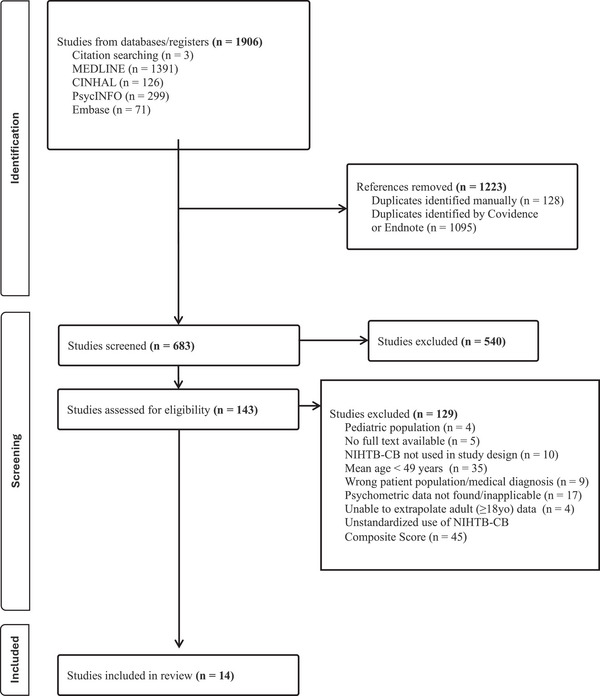
PRISMA flowchart outlining the screening strategy used to identify relevant studies. An initial search of MEDLINE, Embase, PsycINFO, and CINHAL yielded 1906 records. After removing duplicates and applying initial screening criteria, 143 full‐text articles were retrieved for detailed eligibility assessment. Studies were excluded if they reported a mean sample age < 49 years, analyzed pediatric or younger adult data without separate age groups, lacked applicable psychometric data on the NIHTB‐CB's measurement properties, or presented only individual subtest scores without composite scores. Fourteen studies met the inclusion criteria and were included in this review. NIHTB‐CB, National Institutes of Health Toolbox Cognition Battery; PRISMA, Preferred Reporting Items for Systematic Reviews and Meta‐Analyses.

Covidence Systematic Review Software^24^ was used to streamline the production of this systematic review. Figure 1 outlines the selection process. Duplicates were removed as a function of the Covidence software, and initial abstract screenings were conducted to remove irrelevant studies. Study eligibility was assessed by independent, full‐text reviews from at least two reviewers (A.B.T., A.L.M., and/or C.B.L.). Any disagreements regarding inclusion were resolved by consensus with the third reviewer. Data on the patient population in each sample were then extracted by two reviewers (A.W. and O.B.; see Table 2). One reviewer (C.B.L.) recorded the psychometric properties assessed in the studies in the data extraction table, with a second reviewer (A.B.T.) completing an independent review of the measurement properties to ensure data were not missed.

**TABLE 2 alz70673-tbl-0002:** Study characteristics of articles in review.

		Population characteristics				Characteristics of NIHTB‐CB administration		
Study	Study aim	*N*	Age years, *M* (SD)	Sex, female (%)	Education years, *M* (SD)	Target population	Language	Reported composite scores
Brewster et al.^25^	Compare cognitive performance (i.e., NIHTB‐CB Total, Fluid, and Crystallized composites) of caregivers for a person with ADRD to the demographically adjusted normed NIHTB‐CB	28	65.1 (10.1)	82.1	15.1 (2.5)	Healthy adults	English	Total Fluid Crystallized
Carlozzi et al.^26^	Examine construct validity to support the clinical utility of the NIHTB‐CB in individuals with a stroke	131	57.5 (12.6)	49.0	13.5 (2.4)	Participants with a stroke diagnosis	English	Total Fluid Crystallized
Casaletto et al.^12^	Create fully demographically corrected scores (English version of NIHTB)	1038	49.1 (18.6)	53.3	14.0 (2.6)	Healthy adults	English	Total Fluid Crystallized
Casaletto et al.^27^	Create a fully demographically corrected (Spanish version of NIHTB)	408	44.1 (16.7)^a^	58.1	10.7 (4.3)	Healthy adults	Spanish	Total Fluid Crystallized
Hackett et al.^28^	Investigate the validity and utility of NIHTB‐CB in a clinical setting with patients across the continuum of cognitive decline	247	61.0 (14.7)	48.0	15.6 (1.2)	Healthy adults, Participants with subjective cognitive decline, MCI, Mild dementia, and AD	English	Crystallized
Heaton et al.^11^	Investigate the psychometric properties of the NIHTB‐CB composite scores in the adult sample	268	52.3 (21.0)	55.6	13.4 (2.9)	Healthy adults	English	Total Fluid Crystallized
Karpouzian‐Rogers et al.^29^	Examine differences in the NIHTB‐CB between Super Agers and those with average‐for‐age cognition	Total = 77 Super Agers^b^, ^c^, = 46 Cognitively normal 80 year old = 31	84.2 (3.3)	66.2	Super Agers = 17.2 (2.1) Cognitively normal = 16.5 (2.0)	Healthy adults	English	Total Fluid Crystallized
Ma et al.^30^	Investigate the latent factor structure of NIHTB‐CB and its measurement invariance across clinical diagnosis and key demographic variables for the AD research sample	411	66.3 (9.8)	58.4	15.8^c^	Healthy adults, Participants with MCI or dementia, AD	English	Fluid Crystallized
MacAulay et al.^31^	Investigate psychometric properties of NIHTB‐CB compared to corresponding gold‐standard measures in terms of sociodemographic characteristics and technology use	121	70.69 (6.5)	73.6	15.6 (2.7)	Healthy adults	English	Fluid Crystallized
Macaulay et al.^32^	Estimate the effects of high‐intensity strength training on fluid cognition using the NIHTB‐CB	20	69.1 (5.8)	70.0	Not given	Healthy adults	English	Fluid Crystallized
Nitsch et al.^33^	Compare uncorrected versus demographically corrected NIHTB‐CB score types in individuals with TBI and stroke	Total = 789 TBI = 184 Stroke = 211	TBI/controls = 39.1–39.5 (17.0–17.1) Stroke/controls = 56.1–56.5 (13.0–13.3)	TBI/controls = 36.4 Stroke/controls = 43.2	TBI = 13.7 (2.5) TBI‐controls = 14.0 (2.7) Stroke = 13.7(2.6) Stroke‐controls = 13.9 (2.5)	Healthy adults Participants with TBI or stroke	English	Fluid Crystallized
Parsey et al.^34^	Demonstrate the utility of the iPad version of the NIHTB‐CB	51	64.29 (6.7)	62.7	16.6 (1.9)	Healthy adults	English	Total Fluid Crystallized
Scott et al.^35^	Investigate the psychometric properties (test–retest reliability, convergent/discriminant validity) of the NIHTB‐CB	61	67.73 (5.3)	65.6	16.4 (2.5)	Healthy adults	English	Fluid Crystallized
Tyner et al.^36^	Understand how symptoms cluster together, within and across domains of MCI and dementia using the NIHTB	165	75.73^d^	41.2	16.3^d^	Participants with MCI or dementia, AD	English	Fluid Crystallized

*Note*: Proxy data to calculate average education years were high school (12.0), some college (14.0), 4‐year college/bachelor's degree (16.0), Greater than college/master's degree (18.0), and doctorate (20.0).

Abbreviations: AD, Alzheimer's disease; ADRD, Alzheimer's disease and related dementias; GED, General Educational Development test; MCI, mild cognitive impairment; NIHTB‐CB, National Institutes of Health Toolbox Cognition Battery; SD, standard deviation; TBI, traumatic brain injury.

^a^ Paper met exclusion criteria for mean age being < 49 years; however, the article was retained because the study added value in the overall discussion of the NIHTB in Spanish‐speaking adults.

^b^ Super Agers refers to individuals > 80 with exceptional memory, performing as well as or better than average 50‐ to 60‐year‐olds.

^c^ Education years estimated by the review team based on the summary of participant education years by subgroup (e.g., high school/GED, bachelor's degree, etc.). The less‐than‐high‐school/GED subgroup was not included in the estimated average.

^d^ Average age estimated by review team using class midpoints for frequency distribution provided in article.

#### 2.1.4 Assessing risk of bias and evaluation of measurement property results

Two reviewers (A.B.T. and A.L.M.) independently evaluated the methodological risk of bias in measurement properties of the included studies using the COSMIN Risk of Bias checklist. As outlined by COSMIN, the risk of bias in the measurement properties for each study was evaluated using a consistent 4‐point scale, with the lowest rating determining the final risk of bias within each measurement property. Disagreements between the two reviewers were resolved by consensus with a third reviewer (C.B.L.). Consensus with a third reviewer was guided by the user manual for COSMIN methodology^14^ which provided more specific instructions for each rating category. Results of each individual study were then evaluated using the COSMIN criteria for good measurement properties, with the available ratings ranging from “sufficient (+),” “insufficient (−),” or “indeterminate (?).”^14^, ^37^ Criteria were expanded as COSMIN criteria did not include results for exploratory factor analysis (EFA) for structural validity or use of Pearson correlation coefficients for test–retest reliability. Our team followed the criteria outlined in a systematic review that adhered to COSMIN guidelines, including a “sufficient (+)” rating for an EFA with at least 50% of the variance explained by the factors and Pearson correlation coefficients ≥ 0.80 for reliability.^38^ As proposed by COSMIN guidelines, a priori hypotheses were applied by the reviewers to evaluate hypothesis testing for construct validity (convergent, discriminant, and known groups validity). These forms of evidence also contribute to the extrapolation inference used to build a validity argument within the IUA framework^15^, ^16^, ^17^ (see next section below). Our a priori hypotheses for strong support of the psychometric properties of NIHTB‐CB for individuals with ADRD were as follows.

1. *r* ≥ 0.50 for correlations with comparator instruments measuring similar constructs to the NIHTB‐CB.
2. Correlations with instruments measuring related but dissimilar constructs to the NIHTB‐CB were lower (i.e., 0.30–0.50).
3. Correlations with instruments measuring constructs unrelated to the NIHTB‐CB were hypothesized to be < 0.30.
4. Correlations outlined under hypotheses 1, 2, and 3 differed by a minimum of 0.10.
5. Score differed between relevant groups (e.g., populations with MCI diagnoses vs. without an MCI diagnosis).

Results from all studies were categorized by each measurement property and qualitatively summarized as “sufficient (+),” “insufficient (−),” “inconsistent (±),” or “indeterminate (?)”.^14^ Summarized evidence for construct validity was rated as “sufficient (+)” if ≥ 75% of the results aligned with either the a priori hypotheses or the hypotheses outlined in the study and “insufficient” if < 75% of the summarized results failed to align with the hypotheses. The qualitative summary included ranges of values and percentages of supported hypotheses for each measurement property.

### 2.2 Argument‐based approaches to validation: The IUA framework

The IUA framework is a structured approach used to evaluate the validity of assessment outcomes.^15^ This framework emphasizes the importance of articulating the connections between assessment scores and their intended interpretations and uses.^16^ Using this framework, all assessment activity has the same conceptual stages: (1) observing performance on specific tasks (e.g., multiple‐choice test, procedural task), (2) deriving a score from this observed performance that is assumed to accurately reflect the level of performance, (3) combining several scores to generate an overall score or interpretation that is assumed to reflect the desired performance in test settings,; (4) assuming the performance in a test setting reflects the desired performance in a real‐life settings, and (5) further assuming the performance constitutes a rational basis for making a meaningful decision.^15^, ^16^, ^17^ Each of these assumptions is an inference that may not be supported by the evidence. Ultimately, the IUA framework aims to ensure assessments accurately reflect the knowledge and skills they intend to measure, thereby supporting informed decision making.^15^ Protocols for reviewing and evaluating all articles were guided by the COSMIN framework and methodology, and language from the IUA framework is integrated throughout the article to aid in the interpretability of the results and conclusion. Definitions of the integrated language on validity inferences within the IUA framework (scoring, generalization, extrapolation, and implications/decisions) and their corresponding COSMIN taxonomy are provided in [Table alz70673-tbl-0002]Table 3.

**TABLE 3:** Validation inferences in the IUA framework and associated COSMIN taxonomy.

Validity inference	Assumption^a^	Measurement properties (COSMIN taxonomy)
Scoring	Score from an observation accurately captures key elements of performance	Structural validity Cross‐cultural validity/measurement Invariance
Generalization	Total score reflects performance across the test domain	Internal consistency Reliability
Extrapolation	Total score represents meaningful performance in a real‐life setting	Measurement error Criterion validity Hypothesis testing for construct validity Responsiveness
Implications/decisions	Measured performance comprises a rational basis for meaningful decisions and actions	*Not defined within the COSMIN framework*

*Note*: See Cook et al.^16^, ^17^ and Kane^15^ for details and examples.

Abbreviations: COSMIN, Consensus‐based Standards for the Selection of Health Measurement Instruments; IUA, Interpretation/Use Argument.

^a^ Assumptions about the creation and use of assessment results underpin each inference.

### 2.3 Grading quality of evidence

The quality of evidence for each measurement property was graded using a modified version of the “Grading of Recommendations Assessment, Development, and Evaluation” approach.^14^ Within the COSMIN framework, evidence quality is graded as high, moderate, low, or very low by factoring the risk of bias (i.e., methodological quality), inconsistency of results across studies, imprecision (i.e., total sample size of included studies), and indirectness of results.^14^ For this review, the terms “exemplary,” “proficient,” “developing,” and “missing” were used to improve interpretability of the results. Complete definitions of the evidence quality level are provided below.

1. Exemplary evidence (i.e., COSMIN term: high quality) should be interpreted as the review team is very confident the true measurement property lies close to that of the estimate (i.e., pooled/summarized results) of the measurement property.
2. Proficient evidence (i.e., COSMIN term: moderate quality) should be interpreted as the review team is moderately confident in the measurement property estimate; the true measurement property is likely to be close to the estimate of the measurement property, but there is a possibility that it is substantially different.
3. Developing evidence (i.e., COSMIN term: low/limited quality) should be interpreted as confidence in the measurement property estimate is limited: the true measurement property may be substantially different from the estimate of the measurement property.
4. Missing evidence (i.e., COSMIN term: very low quality) should be interpreted as our review team having very little confidence in the measurement property estimate. This does not necessarily mean the information does not exist. Instead, it means the evidence was not documented in the article.^14^

Three authors (A.B.T., C.B.L., and A.L.M.) independently performed the above processes from the risk of bias assessment to grading the quality of evidence (i.e., our confidence in measurement property estimates), with all three authors convening to produce the final consensus of the results.

## 3 RESULTS

Our database search yielded 1906 articles. Duplicates were removed, and 683 articles were screened for relevance based on their titles and abstracts. One hundred forty‐three articles remained after abstract screening. Liberal retention of articles for full‐text review was undertaken to ensure that relevant data were not missed. After full‐text review and analysis of how NIHTB‐CB composites were used within studies, 129 studies were excluded. The most frequent reason for exclusion was lack of standardized use of composite scores (*n* = 45), followed by the mean age of the study sample < 49 years (*n* = 35). The absolute agreement rate between the two independent reviewers was 77.0% (Cohen kappa *=* 0.678, 95% confidence interval: 0.667–0.693) before consensus with a third reviewer, with score ratings exhibiting 88.9% adjacent agreement (i.e., reviewers’ scores stayed within 1 point of each other) across all 70 measurement property ratings for the three composite scores. This process resulted in 14 articles^11^, ^12^, ^25^, ^26^, ^27^, ^28^, ^29^, ^30^, ^31^, ^32^, ^33^, ^34^, ^35^, ^36^ describing the measurement properties of the NIHTB‐CB composite scores for older adults. Per COSMIN guidelines,^14^ each composite should be rated separately; therefore, Fluid, Crystallized, and Total composite scores were assumed to represent separate measures that required separate ratings. Seven articles^11^, ^12^, ^25^, ^26^, ^27^, ^29^, ^34^ reported Fluid, Crystallized, and Total composite scores, which required three separate ratings of the evidence for each summary score. Six additional articles^30^, ^31^, ^32^, ^33^, ^35^, ^36^ required two ratings, as only Fluid and Crystallized composites were reported. The last article^28^ only reported Crystallized composite scores and was rated once.

### 3.1 General characteristics of articles

Characteristics of the included articles are presented in Table 2. Almost all included articles used the English version of the NIHTB‐CB; only one article^27^ used the Spanish version. The article that used the Spanish version of the NIHTB‐CB reported a mean sample age of 44.1; however, the article was retained, despite the mean study sample age meeting exclusion criteria, due to its unique and foundational contribution to understanding the measurement properties of the NIHTB‐CB in a non–English‐speaking population. This study offered valuable preliminary insights into the performance of the NIHTB‐CB within a Spanish‐speaking context, underscoring the critical need to develop and validate culturally and linguistically appropriate cognitive assessment tools for the growing Spanish‐speaking older adult population, thereby guiding future ADRD research with the NIHTB‐CB. Due to the sample's mean age of 44.1 years, the generalizability of the Spanish version of the NIHTB‐CB's performance findings to older adult populations is notably limited. One study^32^ did not provide demographic data on education. For all studies that provided years of education data, the average was ≈ 14.9 years. Only three studies^28^, ^30^, ^36^ included individuals with cognitive decline (e.g., MCI and dementia attributed to AD), two studies^26^, ^33^ included individuals with a history of stroke or TBI, and all other studies included healthy older adults (*n* = 9).

### 3.2 Synthesized evidence

Table 4 presents the overall ratings of the evidence for each measurement property of the NIHTB‐CB, and the quality of the evidence is described below. For the measurement property of content validity, attention should be given to an article discussing a geriatric expert working group's review of the NIHTB content, presentation, and administration for relevancy and applicability in a geriatric population^39^; however, there is no description of the comprehensiveness of the Cognitive Battery within the article (per COSMIN guidelines). Thus, content validity was not assessed. No studies addressed the COSMIN‐specific taxonomy of “responsiveness”; thus, the review could not assess “responsiveness.”

**TABLE 4:** Summary of review findings.

Composite	Measurement property	Summary or pooled evidence	Overall rating	Evidence
Total	Reliability	Sufficient ICC and Pearson correlation of studies^11^, ^34^ Total sample size 319	Sufficient (+)	Exemplary
Total	Criterion validity	Correlation between NIHTB‐CB and GS^11^: 0.89 Total sample size 268	Sufficient (+)	Exemplary^a^
Total	Construct validity: convergent/discriminant validity	Demonstrated good convergent validity with GS^11^: 0.89	Sufficient (+)	Exemplary
Total	Construct validity: known‐groups validity	Qualitative summary for known‐groups validity: Caregivers of PLwD^25^: 1 out of 1 supported (100%) Super Agers^29^: 1 out of 1 supported (100%) Total = 100%	Sufficient (+)	Exemplary
Fluid	Structural validity	Two‐factor structure for aged > 65 years^30^ Three‐factor structure for < 65 years^30^ Two Fluid Tests too challenging for the AD/MCI group led to high missing rates^30^ CFA demonstrated good model fit^30^ *N* = 411	Sufficient (+)	Developing; More research needed for the AD/MCI population
Fluid	Internal consistency	Cronbach alpha values^11^, ^31^: 0.615–0.83 *N* = 389	Inconsistent (±)	Proficient; Inconsistent results;
Fluid	Cross‐cultural validity/measurement invariance	Important differences noted between group factors are less sensitive for MCI/dementia^30^ *N* = 411	Insufficient (–)	Developing; More research needed for AD/MCI population
Fluid	Reliability	Sufficient ICC,^11^, ^32^ CCC,^35^ and Pearson correlations^34^ *N* = 400	Sufficient (+)	Exemplary; consistent results
Fluid	Measurement error	Poor precision noted, no systematic bias^35^ *N* = 61	Insufficient (–)	Developing; *N* < 100
Fluid	Criterion validity	Correlations between NIHTB‐CB and GS^11^, ^35^: 0.58–0.78 *N* = 329	Inconsistent (±)	Proficient; Inconsistent results
Fluid	Construct validity: convergent/discriminant validity	Convergent validity with GS^11^, ^31^: 0.78–0.799 Discriminant validity: 0.19–0.231^31^ ^,^ ^b^, 0.662^31^ ^,^ ^c^	Inconsistent (±)	Proficient; Inconsistent results
Fluid	Construct validity: known‐groups validity	Qualitative summary for known‐groups validity: Caregivers of PLwD^25^: 1 out of 1 (100%) Super Agers^29^: 1 out of 1 (100%) Mild versus mod/severe stroke^26^: 1 out of 1 (100%) TBI/stroke^33^: 1 out of 1 (100%) Total: 100%	Sufficient (+)	Exemplary
Crystallized	Structural validity	Two‐factor structure for aged > 65 years^30^ Three‐factor structure for < 65 years^30^ CFA demonstrated good model fit^30^ Two‐factor solution explains 72% of variance^28^ Two‐factor structure for NIHTB‐CB^36^ ^,^ ^d^ *N* = 823	Inconsistent (±)	Proficient
Crystallized	Internal consistency	Cronbach *α* = 0.84–0.91^11^, ^31^ *N* = 389	Sufficient (+)	Exemplary
Crystallized	Cross‐cultural validity/measurement invariance	Measurement invariance was generally confirmed^30^ *N* = 411	Sufficient (+)	Exemplary
Crystallized	Reliability	Sufficient ICC,^11^, ^32^ CCC,^35^ Pearson correlations^34^ *N* = 400	Sufficient (+)	Exemplary
Crystallized	Measurement error	Poor precision, no systematic bias^35^ *N* = 61	Insufficient (–)	Developing; *N* < 100
Crystallized	Criterion validity	Correlations between NIHTB‐CB and GS^11^, ^35^ = 0.84–0.90 *N* = 329	Sufficient (+)	Exemplary
Crystallized	Construct validity: convergent/discriminant validity	Convergent validity with GS^11^, ^31^: 0.78–0.799 Discriminant validity: 0.273–0.39,^31^ ^,^ ^b^ 0.662^31^, ^c^	Inconsistent (±)	Proficient; Inconsistent results
Crystallized	Construct validity: known‐groups validity	Qualitative summary for known‐groups validity: Care givers of PLwD^25^: 1 out of 1 (100%) Super Agers^29^: 1 out of 1 (100%) Mild versus mod/severe stroke^26^: 1 out of 1 (100%) TBI/stroke^33^: 1 out of 1 (100%) Total = 100%	Sufficient (+)	Exemplary

Abbreviations: AD, Alzheimer's disease; CCC, concordance correlation coefficients; CFA, confirmatory factor analyses; GS, gold standard measures; ICC, intraclass correlation; NIHTB‐CB, National Institutes of Health Toolbox Cognition Battery; MCI, mild cognitive impairment; PLwD, person living with dementia; TBI, traumatic brain injury.

^a^ One study of very good quality; one study of inadequate quality was not included.

^b^ Level of education for the sample reported as college or greater

^c^ Level of education for the sample reported as high school.

^d^ Six‐factor solution determined to be the most interpretable solution for the entire NIHTB collection (i.e., all cognitive, motor, sensory, emotional, and social measures). Two‐factor solution (Fluid Intelligence and Crystallized Intelligence) found for NIHTB‐CB.

### 3.3 Scoring inference

The evidence provided by the measurement property of “structural validity” and “cross‐cultural validity/measurement invariance” in the COSMIN framework aligns with the “scoring inference” in an IUA validation framework.

#### 3.3.1 Structural validity

Three studies assessed the structural validity of the NIHTB‐CB. Any articles that did not identify the latent factor framework of the Total, Fluid, or Crystallized composite scores were excluded. Two studies included participants with cognitive decline (e.g., MCI, dementia due to AD) and healthy older adults.^28^, ^30^ Ma et al.^30^ and Hackett et al.^28^ noted that two Fluid subtests of the NIHTB‐CB (i.e., Picture Sequence Memory and List Sorting Working Memory) were more difficult for the MCI/AD group. Tyner et al.^36^ included only individuals with a diagnosis of amnestic MCI or “mild dementia of the Alzheimer's type.” All three studies assessing structural validity reported increased missing data for these two subtests compared to the other subtests from the groups with MCI/dementia due to AD. While Hackett et al. did not provide percent missingness for the two subtests, the other studies reported missing data ranging from 18%^36^ to 40.2%^30^ for Picture Sequence Memory and 9.7%^36^ to 17.1%^30^ for List Sorting Working Memory tests. Tyner et al.^36^ posited that higher missing data may be from the discontinuation criterion requiring successful practice item completion. Fatigue from prolonged cognitive testing might also contribute, as both studies administered the NIHTB‐CB alongside other cognitive measures.

Differences in structural validity evaluation were noted. Ma et al.^30^ evaluated the Fluid and Crystallized composites by analyzing all seven tests (i.e., two Crystallized and five Fluid) in cognitively impaired older adults. An EFA on the whole sample yielded a good‐fitting two‐factor solution with distinct Fluid and Crystallized factors. The two‐factor model was confirmed by confirmatory factor analysis (CFA), showing good fit across all groups except those aged < 65 years with slight variations: (1) Working Memory demonstrated small cross‐loadings on the Crystallized cognition factor for the cognitively unimpaired (0.24) and non–under‐represented groups (0.19), and (2) the residual variance of Reading was set to zero for model identification needs for the cognitively impaired group. A three‐factor solution (i.e., executive functioning/processing speed, memory, and language) was found for the group aged < 65 years. However, performing both EFA and CFA on the same sample may have inadvertently capitalized on chance, increasing the risk of overfitting the model.

Hackett et al.^28^ omitted two challenging tests (i.e., Picture Sequence Memory and List Sorting Working Memory) for the combined MCI/dementia due to AD group. They analyzed the two Crystallized, three Fluid subtests (Dimensional Change Card Sort, Flanker Inhibitory Control/Attention, and Pattern Comparison Processing Speed), and two supplemental NIHTB‐CB measures (i.e., Rey Auditory Verbal Learning Task immediate recall trials 1–3, Rey Auditory Verbal Learning Task delayed recall) were analyzed. Standard NIHTB‐CB Fluid composite scoring was not used, so only the structural validity of the Crystallized composite could be rated for this review. Principal component analysis (PCA) was performed to identify the factor structure of the uncorrected NIHTB‐CB scores. Three separate PCAs were conducted: (PCA 1a) on the included NIHTB‐CB tests (i.e., two Crystallized and three Fluid) and the supplemental tests, including the Rey Auditory Verbal Learning Task delayed recall; (PCA 2a) on the included NIHTB‐CB tests (i.e., two Crystallized and three Fluid) and the supplemental scores, excluding the Rey Auditory Verbal Learning Task delayed recall; and (PCA 3) on traditional tests of cognitive function. For the extent of this review, our team focused on PCA 2a, as this PCA had the fewest added supplemental tests. PCA 2a identified a solution with only two factors explaining 72% of the variance, including (1) executive function/working memory and (2) Crystallized intelligence. Both Crystallized tests (i.e., Oral Reading Recognition and Picture Vocabulary) included in PCA 2a demonstrated large, positive factor loadings (0.70–0.89) for the Crystallized intelligence construct. While PCAs can be useful for reducing dimensionality, they may not provide the depth of understanding needed to identify and interpret latent factors effectively.

Tyner et al.^36^ evaluated the structural validity of all cognitive, motor, sensory, emotional, and social measures of the NIHTB‐CB using EFA to detect symptom clusters for MCI and dementia of the AD type. A six‐factor solution was the most interpretable solution for the entire NIHTB collection. All seven measures from the NIHTB‐CB loaded onto a clear latent framework for the Fluid (Factor 1: Fluid Intelligence) and Crystallized (Factor 2: Crystallized Intelligence) composites within this model. All five Fluid subtests positively loaded onto Factor 1 (Fluid Intelligence). However, one motor and one sensory subtest exhibited weak‐to‐moderate correlations (0.35–0.43) with the Fluid Intelligence factor, aligning with the study's aim. As these additional subtests loaded onto the Fluid Intelligence factor, only the structural validity of the Crystallized Composite was evaluated. Both Crystallized subtests demonstrated moderate‐to‐large factor loadings, with Picture Vocabulary (rounded to 1.00) dominating Factor 2 compared to Oral Reading Recognition (0.62). Ma et al.^30^ also observed Picture Vocabulary (0.93) dominating the Crystallized factor in the MCI/dementia group.

The methodological quality of the study by Ma et al.^30^ demonstrating the structural validity of the Fluid composite was rated as good according to the COSMIN guidelines. Evidence evaluating the structural validity of the Fluid composite score was rated as developing. There was evidence of indirectness in the results (e.g., some Fluid tests were considered too challenging, and there was a different/three‐factor model for the group aged < 65 years); however, concerns arose regarding potential issues with model overfitting and misspecification. Findings indicate the true structural validity of the Fluid composite may substantially differ from the current estimates found in the literature for these populations (i.e., the structural validity of the Fluid composite score needs to be confirmed and requires a broader research perspective, incorporating multiple studies and diverse methodologies). Thus, increased efforts are needed to study the performance of the NIHTB‐CB in older and ADRD populations. The methodological quality of the studies that demonstrated the structural validity of the Crystallized Composite was summarized with a COSMIN rating of at least adequate quality.^28^, ^30^, ^36^ The summarized evidence was rated as proficient. Thus, the pooled evidence is likely to be close to the estimate of the true structural validity of the Crystallized composite score.

While other articles assessed the structural validity of the NIHTB‐CB, they were excluded based on our review criteria. Notably, one such excluded study, by Nolin et al.,^40^ provided robust psychometric findings with key aspects of interest for future NIHTB‐CB research. This study observed correlations between uncorrected Crystallized subtests (*r* = 0.641) and their relation to uncorrected Fluid intelligence factors (e.g., Vocabulary and Episodic Memory inter‐correlation = 0.537)^40^. Given these findings, the authors stated that no clear separation of Crystallized/Fluid factors was identified. Arguably, correlations in the range of 0.5 to 0.6 could also provide evidence that Crystallized and Fluid are related yet distinct factors. However, as the authors stated the Fluid and Crystallized composites did not distinctly map onto the latent factors identified; the article did not meet the inclusion criteria of needing to present findings on the Crystallized and Fluid composites.

Nolin et al^40^. notably found a six‐factor model (Vocabulary, Reading, Episodic Memory, Working Memory, Executive Function, and Speed) best fit fully corrected NIHTB‐CB scores in cognitively unimpaired adults aged 85+ and demonstrated convergent and discriminant validity consistent with these domains. Consistently high inter‐correlations observed between executive function and other domains prompted Nolin et al^40^. to suggest this finding underscores the challenge of classifying tests to a single domain, given that subtest performance is likely influenced by limitations in other domains.^40^

#### 3.3.2 Cross‐cultural validity/measurement invariance

Ma et al.^30^ assessed measurement invariance using multi‐group confirmatory factor analyses. Invariance testing was conducted for subgroups based on diagnosis (i.e., cognitively unimpaired, MCI/dementia), sex (i.e., female, male), race/ethnicity (i.e., under‐represented groups, non–under‐represented groups), age (i.e., < 65 years, ≥ 65 years), and education (i.e., without bachelor's degree vs. with bachelor's degree/high school education). Ma et al.^30^ assessed measurement invariance based on a two‐factor structure (Crystallized and Fluid latent factors) except for age, which demonstrated a three‐factor model for the younger than age 65 group. The three‐factor model further separated Fluid cognition into two factors (i.e., executive function and processing speed) for the group < 65 years. Results confirmed configural invariance across diagnosis subgroups, with both Fluid and Crystallized cognition factors present in both the cognitively impaired and unimpaired groups. However, partial metric and scalar invariance were found across diagnosis subgroups. Specifically, the Episodic Memory test yielded a greater loading within the Fluid Cognition factor for the cognitively unimpaired group than the group with dementia/MCI. This finding was interpreted as suggesting that the test was “less sensitive” in detecting individual Fluid cognition differences for the cognitively impaired group. Moreover, both Episodic Memory and Working Memory tests yielded higher indicator intercepts for the cognitively unimpaired. This result implied these two tests were more difficult for individuals in the dementia/MCI group, which is to be expected. Thus, a finding that the memory‐related tests are somewhat less strongly related to the other Fluid tests in these particular groups does not imply that these former tests are less accurate or valuable for such groups. Importantly, it was noted that Episodic Memory and Working Memory subtests also had more missing data from the group with dementia/MCI because they were too challenging for participants with cognitive impairment and yielded low completion rates. At the scalar invariance level, measurement invariance was generally confirmed across sex, race/ethnicity, and education, allowing for meaningful comparisons of latent factor means, variances, and correlations of demographic differences across these subgroups.

Due to different loadings of some Fluid cognition differences for the cognitively impaired group, the Fluid composite results would suggest developing evidence in its pure, psychometric quality (i.e., the true property of measurement invariance may be substantially different from the estimate provided). This does not mean that the group of tests contained in the Fluid Composite are failing to accurately reflect cognitive pattern differences caused by ADRD. Instead, findings reflect a need for increased efforts to study the NIHTB‐CB in ADRD populations, as only one study provided evidence for measurement invariance in this population. The Crystallized composite evidence was rated as exemplary. Scalar invariance was confirmed across group variables, and no important group differences for the Crystallized composite were noted in the study.

### 3.4 Generalization inference

The evidence provided by the measurement property of “internal consistency” and “reliability” in the COSMIN framework aligns with the “generalization inference” in an IUA validation framework.

#### 3.4.1 Internal consistency

The internal consistency reliability of both the NIHTB‐CB Crystallized composite score and Fluid composite score was assessed by two studies with very good methodological quality.^11^, ^31^ The studies demonstrated varied results for the reliability of the Fluid composite score. Coefficient alpha of the Fluid composite score from the Heaton et al. study demonstrated adequate internal consistency reliability for research purposes (*α* = 0.83),^11^ while MacAulay et al.^31^ reported lower internal consistency reliability for the Fluid composite (*α* = 0.615). Differences in group demographics were therefore explored in both studies. Both studies demonstrated approximately the same range of income diversity in their samples. Average years of education in the MacAulay et al.^31^ and Heaton et al.^11^ studies were reported as 15.6 and 13.4, respectively. Samples in both studies included healthy individuals; most participants in the MacAulay et al.^31^ sample identified as non‐Hispanic White (99.2%) compared to the sample assessed by Heaton et al.^11^ (non‐Hispanic White = 55.2%). Therefore, the studies were pooled together, and the quality of evidence for internal consistency reliability of the Fluid composite score was rated as proficient for research purposes.

For the Crystallized composite score, values of coefficient alpha in both the Heaton et al.^11^ and MacAulay et al.^31^ studies demonstrated high internal item consistencies and were > 0.80 (*α* = 0.84–0.91). Both studies of the NIHTB‐CB Crystallized composite scores demonstrated very good methodological quality for internal consistency. The overall rating for the summarized Crystallized composite score reliability results was sufficient (+) for research purposes and individual decisions. The quality of evidence for the internal consistency of the Crystallized composite score was rated as exemplary based on the summarized results. Future research on the internal consistency of NIHTB‐CB composite scores should explore omega estimates. Coefficient alpha is a good starting point for internal consistency reliability estimates, but alphas are based on a restrictive psychometric model and assumptions. Thus, coefficient omega may provide more robust internal consistency reliability estimates.^41^, ^42^

#### 3.4.2 (Test–retest) reliability

Test–retest reliability (i.e., “Reliability” per COSMIN‐specific taxonomy) of the NIHTB‐CB was assessed in four studies^11^, ^32^, ^34^, ^35^ of adequate methodological quality. Two studies^11^, ^34^ reported test–retest reliability statistics for all composite scores (i.e., Total, Fluid, Crystallized). The two other studies^32^, ^35^ reported test–‐retest reliability statistics for only Fluid and Crystallized composite scores. Test–retest reliability was evaluated by Pearson correlation coefficients,^34^ intraclass correlation coefficients (ICC),^11^, ^32^ and concordance correlation coefficients (CCC).^35^ The review team used the COSMIN guidelines of ICC ≥ 0.70 to rate both the ICC and CCC for reliability. As previously noted, the review team also expanded the COSMIN criteria to indicate Pearson correlation coefficients ≥ 0.80 to be rated as “sufficient.”

For the Total composite score, test–retest reliability estimates were sufficient, with the Heaton et al. study^11^ evaluating test–retest reliability with an ICC (0.86) and the Parsey et al.^34^ study using a Pearson correlation coefficient (*r* = 0.90). For the Fluid composite, summarized evidence was sufficient, with Heaton et al.^11^ and Macaulay et al.^32^ evaluating test–retest reliability with ICCs (Heaton et al.^43^: 0.79; Macaulay et al.^32^: 0.77), Scott et al.^35^ using a CCC (0.73), and Parsey et al.^34^ using a Pearson correlation coefficient (*r* = 0.83). Last, test–retest reliability estimates for the Crystallized composite score were sufficient, with Heaton et al.^11^ and Maculay et al.^32^ evaluating test–retest reliability with ICCs (Heaton et al.^11^: 0.92; Macaulay et al.^32^: 0.91), Scott et al.^35^ using a CCC (0.92), and Parsey et al.^34^ using a Pearson correlation coefficient (*r* = 0.90). The quality of evidence for the test–retest reliability of the Total, Fluid, and Crystallize composite scores was all rated as exemplary based on consistent results.

### 3.5 Extrapolation inference

The evidence provided by the measurement property of “measurement error,” “criterion validity,” “hypothesis testing for construct validity,” and “responsiveness” in the COSMIN framework aligns with the “extrapolation inference” in an IUA validation framework.

#### 3.5.1 Measurement error

Per COSMIN guidelines,^14^ “measurement error” describes the error in a participant's score that is not due to changes in the measured trait. Scott et al.^35^ assessed measurement error (per COSMIN taxonomy) for both the Fluid and Crystallized composites by assessing agreement between the age‐adjusted NIHTB‐CB scores and gold standard cognition measures. Age‐adjusted scores were used to facilitate comparison to the NIHTB because fully adjusted normative data were not available for all gold standard tests selected for the study. The NIHTB‐CB demonstrated good agreement (CCC = 0.70); however, the average pairwise discrepancy approached one standard deviation (SD; root mean square deviation = 12.12), which indicated that individual pairs of scores differed by an average of 12.5 points. The NIHTB‐CB Picture Vocabulary test yielded scores that were 8 to 9 standard score points higher than those from the gold standard reading tests. On the NIHTB‐CB Picture Vocabulary test, an age‐corrected standard score near 100 indicates vocabulary ability that is average for the participant's age level, scores ≈ 115 suggest above‐average ability, and scores ≈ 130 represent superior ability (i.e., representative of the top 2% nationally, based on NIHTB‐CB normative data).^22^ A score of 85 indicates below‐average ability, and a score of ≤ 70 suggests markedly low language ability (i.e., bottom 2% nationally).^22^ Notably, the other NIHTB‐CB Crystallized test, the Oral Reading Recognition Test, produced similar scores with the gold standard equivalent^35^.

The NIHTB‐CB and gold‐standard Fluid Cognition composites demonstrated moderate agreement (CCC = 0.55), and the proximity of the CCC to Pearson correlation (*r* = 0.58) mirrors the finding that the two scales have nearly equivalent means (NIHTB‐CB = 109.93, SD = 14.02; GS = 108.93, SD = 10.38).^35^ However, the average difference between individual pairs of scores was 11.60 points. The limits of agreement suggest that 95% of scores between the NIHTB‐CB and the gold‐standard Fluid composites may vary between −21.83 and +23.84 points.^35^ Scott et al.^35^ conclude the NIHTB‐CB may provide imprecise results but found there is no consistent bias (i.e., neither overestimation nor underestimation of results).

Notably, the sample size of the study (*n* = 61) and the homogeneity of the sample limited the ability of the study to explore whether discrepancies were from demographic differences. Exploratory analyses were performed and indicated that the inconsistency seen in the NIHTB‐CB Crystallized composite was not influenced by any specific demographic differences.^35^ However, preliminary analyses of Fluid composite indicated the NIHTB overestimated Fluid cognition for those with high scores and underestimated Fluid cognition for individuals with lower scores.^35^ Study findings also determined the NIHTB‐CB Fluid composite tends to overestimate Fluid cognition in younger participants and underestimate Fluid cognition abilities in participants who identify as Black.^35^ These results are again preliminary and exploratory. Additionally, only age‐adjusted and unadjusted standard scores were used in the analyses, which could explain these findings. Scott et al.^35^ argue caution is warranted when interpreting the Fluid composite scores that are adjusted only for age, as several factors appeared to contribute to the size and direction of the Toolbox–gold standard discrepancy in scores. The study was given an “indeterminate” rating, as COMSIN guidelines refer to minimal important change to grade this measurement property, which may not be applicable for performance‐based outcome measures used in this context.^44^ Evidence for measurement error was rated as developing, as the conclusions were slightly limited by the small sample size of the study (i.e., <100).

#### 3.5.2 Criterion validity

Per COSMIN guidelines,^14^ “criterion validity” describes the degree to which the scores of a measure align with a gold standard. Criterion validity (as defined by COSMIN) was reported for two studies.^11^, ^35^ The studies differed on the selected gold standard matched test. For the Heaton et al.^11^ study, Crystallized composite tests were matched with gold standard equivalents, including the Wide Range Achievement Test^45^ and the Peabody Picture Vocabulary Test Fourth Edition.^46^ Fluid composites on the NIHTB‐CB were matched with gold standard equivalents, including measures of processing speed (i.e., the average of the Wechsler Adult Intelligence Scale Fourth Edition Coding and Symbol Search subtests)^47^ executive function–inhibitory control (i.e., the Delis‐Kaplan Executive Function System Color‐Word Interference score),^48^ executive function–cognitive flexibility (i.e., Wisconsin Card Sorting Test‐Total Errors),^43^ episodic memory (i.e., average of total learning scores from Brief Visuospatial Memory Test–Revised^49^ and Rey Auditory Verbal Learning Test),^50^ and working memory (i.e., average of Paced Auditory Serial Addition Test –first channel^51^ and WAIS‐IV Letter‐Number Sequencing^47^).

Scott et al.^35^ selected the Trail‐Making Test Part B^52^ for executive function–cognitive flexibility, the Stroop Color Word Test^53^ for executive function–inhibitory control, the Wechsler Memory Scale‐Revised (Digit Span subtest)^47^ for working memory, and an earlier edition of the Wechsler Adult Intelligence Scale (Digit Symbol Substitution subtest^54^) for processing speed. The gold standard battery selected by the Scott et al.^35^ study did not include a measure for receptive vocabulary to serve as a criterion measure for the NIHTB‐CB Crystallized composite and instead chose to match the composite with the American National Adult Reading Test.^55^ Scott et al.^35^ state the correlations provided for the Crystallized composite are exploratory.

All correlations provided by the studies represented concurrent validity coefficients. Heaton et al.^11^ reported the NIHTB‐CB Total composite demonstrated sufficient evidence of criterion validity with its gold standard counterpart (*r* = 0.89). The NIHTB‐CB Fluid composite demonstrated varied results, with the Heaton et al. study^11^ demonstrating sufficient evidence of criterion validity between the gold standard Fluid composite and the NIHTB‐CB (*r* = 0.78) and the Scott et al.^35^ demonstrating insufficient evidence of criterion validity (*r* = 0.58). Differences across the studies include: (1) the sample in Scott et al.^35^ was older (*M* = 67.7 years) compared to the Heaton et al.^11^ sample (*M* = 52.3), (2) Scott et al.^35^ used age‐corrected scores for analyses while Heaton et al.^11^ used uncorrected scores, and (3) the selected gold standard measures for assessing criterion validity varied across studies. Finally, neither study included a clinical sample known to be at risk for cognitive impairment; thus, other indicators of criterion validity (e.g., sensitivity and specificity to cognitive impairment) were not examined.

The NIHTB‐CB Crystallized composite demonstrated consistent results, with both Heaton et al.^11^ and Scott et al.^35^ studies demonstrating sufficient evidence of criterion validity between the gold standard Crystallized composite and the NIHTB‐CB (*r* = 0.84–0.90). The findings demonstrated exemplary evidence of sufficient criterion validity for the Total and Crystallized Composites of the NIHTB‐CB. The quality of evidence for the Fluid composite related to criterion validity was rated as proficient.

#### 3.5.3 Hypotheses testing for construct validity

Per COSMIN guidelines^14^ “hypothesis testing for construct validity” is assessed by examining the consistency of the instrument's scores with hypotheses (e.g., internal relationships, convergent and discriminant validity, or differences between relevant groups) based on the assumption that the instrument accurately measures the intended constructs. Qualitative pooling was used to assess the construct validity of the Total, Fluid, and Crystallized composites of the NIHTB‐CB. Heaton et al.^11^ provided sufficient evidence (*r* = 0.89) for the convergent validity of the Total composite and gold standard measures. Heaton et al.^11^ and MacAulay et al.^31^ provided sufficient evidence for convergent validity (*r* = 0.78–0.79) of the Fluid composite and gold standard measures, as well as the Crystallized composite (*r* = 0.85–0.90). Heaton et al.^11^ provided evidence for discriminant validity, indicated by weaker correlations observed between uncorrected NIHTB‐CB Crystallized and gold standard Fluid cognition composite scores (*r* = 0.39), as well as between uncorrected NIHTB‐CB Fluid and gold standard Crystallized composite scores (*r* = 0.19). MacAulay et al.^31^ replicated the methods used in the Heaton et al.^11^ study and supported their findings with evidence of discriminant validity between the uncorrected NIHTB‐CB Fluid scores and gold standard Crystallized composites scores in college (*r* = 0.19) and graduate education (*r* = 0.23) groups. Additionally, MacAulay et al.^31^ found substantially lower correlations between the uncorrected NIHTB‐CB Crystallized scores and gold standard Fluid Composites scores in the college (*r* = 0.27) and graduate education (*r* = 0.36) groups. However, the uncorrected NIHTB‐CB Crystallized and Fluid composites demonstrated poor discriminant validity (*r* = 0.66 for both Fluid and Crystallized Composites) in the high school education group.^31^ The construct validity of the Total composite for the NIHTB‐CB was rated as sufficient, and the quality of evidence was rated as exemplary. Findings on the quality of evidence demonstrating construct validity for the Fluid and Crystallized composites were rated as proficient. Note should be made that Hackett et al.^28^ also found evidence of convergent validity via non‐parametric partial correlations between NITHB‐CB and traditional test factor scores. These results were not included in the summarized evidence to ensure standardized assessment of convergent/discriminant statistics across studies.

Four studies^25^, ^26^, ^29^, ^33^ evaluated known group differences to support a validity argument for score use. Brewster et al.^25^ compared cognitive performance (i.e., NIHTB‐CB Total, Fluid, and Crystallized composites) of caregivers for a person with ADRD to the demographically adjusted normed NIHTB‐CB. Significant differences were noted between the caregivers’ scores and the norms for both the Crystallized and Fluid composites, with caregivers scoring higher for the Crystallized composite (Cohen *d* = 0.79) and lower than the norms on the Fluid composite (Cohen *d* = 0.87).^25^ Karpouzian‐Rogers et al.^29^ compared the episodic memory of Super Agers (i.e., individuals > 80 years with episodic memory comparable to individuals aged 50; *n* = 46) and cognitively average‐for‐age older adults (*n* = 31). Total, Fluid, and Crystallized composite scores of the NIHTB‐CB were also analyzed. Significant effects were noted only on the Picture Sequence scores, with Super Agers performing better than average‐for‐age older adults, aligning with their a priori hypothesis.^29^ No significant group effects were noted on any of the composite scores.

Two studies^26^, ^33^ compared groups with varying levels of neurological impairment. Carlozzi et al.^26^ assessed known group differences of the NIHTB‐CB Fluid and Crystallized composite in individuals with a confirmed diagnosis of stroke. To determine effect sizes, the average scores for the composite and subtest scores were compared to the average score of the NIH Toolbox normative sample (*N* = 972; *M* = 50; SD = 10).^26^ Standard effect size cutoffs of 0.20, 0.50, and 0.80 were used to indicate small, moderate, and large effects. Carlozzi et al.^26^ found that individuals with moderate/severe stroke performed worse than participants with mild stroke on both the fully corrected Fluid and Crystallized composites of the NIHTB if the analyses did not control for motor function.^26^ Effect sizes, reported as Cohen *d*, for the mild stroke group were −0.64 and −1.64 for the Fluid composite scores. For the Crystallized composite scores, the effect size for the mild stroke group was 0.05 and −0.41 for the moderate/severe stroke group. Significant group differences were noted on all subtests within the two composites, except for Picture Vocabulary and List Sorting, with the moderate/severe stroke group performing worse than those with mild stroke.^26^ However, group differences were non‐significant for NIHTB Fluid tests that rely on speeded motor function (i.e., Pattern Comparison, DCCs, and Flanker), suggesting that group differences were partially confounded with motor function difficulties of the participants.^26^ Crystallized and Fluid known group differences confirmed the hypotheses outlined in the Carlozzi et al.^26^ study.

Nitsch et al.^33^ assessed known group differences by comparing uncorrected and demographically corrected Fluid and Crystallized composite scores in two neurologic samples (i.e., TBI, stroke) and their demographically matched controls (i.e., healthy adults). This study found the TBI and stroke groups performed significantly worse when using both the uncorrected and demographically corrected Fluid composite scores, which aligned with an a priori hypothesis defined by the study. No meaningful differences were noted on the Crystallized composite, which also aligned with one a priori hypothesis. The overall ratings of known group differences to support score use in building a validity argument for the NIHTB‐CB scores were sufficient and exemplary.

#### 3.5.4 Responsiveness

According to COSMIN guidelines,^14^ “responsiveness” is defined as the ability of the instrument to detect change over time. No studies found addressed the responsiveness of the NIHTB‐CB to capture longitudinal cognitive change over time. Findings indicate longitudinal data are needed to evaluate if the NIHTB‐CB can detect change in older populations at risk for ADRD.

### 3.6 Demographic differences

Guidelines outlined by COSMIN do not address norming papers and observations of demographic differences from studies that use methods not included in the measurement invariance section. Thus, these demographic differences will be summarized in this section without ratings, as the amalgamation of these demographic differences adds to the overall discussion of future areas of research for the NIHTB‐CB. Note should be made that three types of scores (i.e., uncorrected standard scores, age‐adjusted standard scores, and fully adjusted scores) are produced for every NIHTB‐CB subtest and composite.

#### 3.6.1 Score relationships with age

Three studies^11^, ^12^, ^31^ found the Total composite scores demonstrated a negative relationship with age (*r* = −0.26 to 0.43) in healthy older adults. Heaton et al.^11^ found the gold standard Total composite demonstrated a similar negative relationship with age (*r* = −0.22). Casaletto et al.^27^ also noted this negative relationship with age for the Total composite on the Spanish version of the NIHTB‐CB (*r* = −0.38). Negative relationships with age were more prominent in the Fluid cognition composite (*r* = −0.35 to 0.68),^11^, ^12^, ^31^ with the gold standard for Fluid cognition mirroring these results (*r* = −0.55).^11^ Nitsch et al.^33^ identified age as the demographic variable with the strongest associations to the uncorrected Fluid score and accounted for 14% to 25% of the variance in Fluid performances in the stroke and TBI groups. In the Spanish version of the NIHTB‐CB, Casaletto et al.^27^ noted the Fluid composite score also steadily declined with age (*r* = −0.50) in the Toolbox normative sample. The Crystallized composite score had slightly more varied results, with two studies finding small to negligible positive relationships between age and the Crystallized composite (*r* = 0.10–0.18), which was comparable to the results of the gold standard Crystallized composite (*r* = 0.14).^11^, ^12^ MacAulay et al.^31^ and Parsey et al.^34^ found no significant age effect for the Crystallized composite. Conversely, the Spanish version of the NIHTB‐CB found the Crystallized composite demonstrated a slight, but significant, negative association with older age (*r* = −0.33 for the 40–85 years age group).^27^ For the Spanish version of the NIHTB‐CB, the generalizability of these age‐related findings is limited, as the mean population age in the included study was 44.1 years.

#### 3.6.2 Score relationships with education, employment, and income

Two studies found significant relationships between education and the NIHTB Total and Fluid composite scores in healthy older adults, with both studies demonstrating a positive relationship between both the Total and Fluid composites and education.^11^, ^12^ One study found education was most strongly correlated with the Crystallized composite (*r* = 0.41),^12^ with results from the Spanish version of the NITHB reflecting this same positive relationship (*r* = 0.31).^27^ Casaletto et al.^27^ reported positive correlations between education and the Spanish version NIHTB‐CB Total (*r* = 0.54) and Fluid (*r* = 0.53) composite scores. Note should be made that education was more strongly associated with Fluid composite scores for Spanish‐speaking individuals; conversely, the Crystallized composite scores demonstrated a stronger association among the English‐speaking cohort for participants administered the Spanish version of the NIHTB‐CB (Spanish: Fluid *r* = 0.53; Crystallized *r* = 0.31; vs. English: Fluid *r* = 0.21; Crystallized *r* = 0.41).^27^ All participants were Spanish speakers living in the United States who reported being either monolingual or multilingual. As previously noted, the mean age of this study population was 44.1 years, which limits the generalizability of these relationships with education to older adult populations.

MacAulay et al.^31^ found significant relationships between education and both the Fluid and Crystallized composite scores. Healthy older adults with a high school education demonstrated lower performance compared to the group with some college education on both composites, with the some college education group displaying similar performance to those in the college degree group.^31^ Moreover, the graduate‐level education group performed significantly better than other education‐specific groups on the Crystallized composite.^31^ Attention should be given to the Heaton et al.^11^ study that found lower scores on all three NIHTB‐CB age‐adjusted composite scores were significantly associated with self‐reported history of learning difficulties in school (i.e., grade repetition, grade failure, special classes/tutoring, and overall academic performance). Similar associations were also found for the gold standard composites (i.e., Total, Fluid, Crystallized).^11^ Nitsch et al.^33^ found education demonstrated the strongest non‐clinical (i.e., unrelated to severity of TBI or stroke severity) association with performance in individuals with TBI and stroke, based on uncorrected Crystallized composite scores. Therefore, individuals with a higher number of years of education performed better on the Crystallized composite, which would interfere with any possible contribution to clinical diagnostics of acquired cognitive impairments.^33^ These findings emphasize the necessity and importance of using demographically corrected NIHTB‐CB scores for diagnostic and interpretation purposes. Heaton et al.^11^ also found that employment status (i.e., participants reporting being employed or retired) was positively associated with Total, Fluid, and Crystallized age‐adjusted scores.^11^ Statistically significant effects of household income were also noted on all three age‐adjusted composite scores.^11^

#### 3.6.3 Score relationships with race

Significant relationships were found between race/ethnicity and the Total, Fluid, and Crystallized composite scores, with overall performance favoring non‐Hispanic White participants.^11^, ^12^ The pattern of race/ethnicity effects was consistent across all NIHTB‐CB subtests and composites. Further, the norming studies posit multiple, complex causes related to these noted differences, including the effects of structural racism and systemic barriers on marginalized groups.^11^, ^12^ Casaletto et al.^12^ noted that the fully corrected NIHTB‐CB scores adjust for each demographic factor and potential confounding effects these factors might have on composite scores. However, the current norming program for the NIHTB‐CB does not allow for interaction terms among demographic variables, and Casaletto et al.^12^ stated that future studies would benefit from the creation of norming programs that can adjust for such possible complex relationships. Nitsch et al.^33^ also found relationships between race and uncorrected Fluid and Crystallized composite scores in adults diagnosed with TBI or stroke, with performance on both composites favoring non‐Hispanic White participants. Once demographic characteristics were applied to Crystallized composite scores, race was no longer significantly associated. Similarly, demographically corrected Fluid composite scores were also minimally associated with race.

#### 3.6.4 Score relationship with language

Relationships with language were also noted in the Spanish version of the NIHTB‐CB^27^; however, the generalizability of these findings is limited by the sample's mean age of 44.1 years. Casaletto et al.^27^ noted performance on the Crystallized composite was associated with all language background factors. Increased Spanish‐speaking frequency and Spanish exposure were associated with higher Crystallized composite scores. Participants who spoke Spanish at home, had Spanish as their first language, and were educated or born outside the United States performed better on the Spanish Crystallized composite. Conversely, language background factors demonstrated the opposite effect on Fluid composite score. Participants who spoke both Spanish and English at home demonstrated better performance on the Fluid composite. Additionally, participants who attended school or were born in the United States performed better on the fully corrected Fluid composite.

## 4 DISCUSSION

This systematic review evaluated 14 studies of the NIHTB‐CB Total, Crystallized, and Fluid composite properties. Many articles (i.e., 45) were excluded due to using individual or additional subtests, administering some (not all) Fluid or Crystallized measures, or not reporting composite scores. The scope of this systematic review was limited to the psychometric properties of the NIHTB‐CB composite scores. Future reviews should investigate the psychometric properties of individual NIHTB‐CB subtests. We also excluded 35 studies in which the mean age was < 49 years, as most research focused on younger populations or combined wide age ranges without stratification. Only six included studies focused on neurologically impaired individuals. Research on the Spanish NIHTB‐CB for older adults is scarce, with a single study on the Spanish version included in this review but the study sample mean was < 49 years. Crucially, no included studies assessed cognitive change within clinical groups (e.g., treatment improvement or disease progression) or reported norms for detecting significant changes over time using test–retest data from a clinically stable population.^56^ Measurement properties of the Total composite were infrequently evaluated, with only seven articles reporting statistics.

Four papers supported the scoring inference for the Crystallized and/or Fluid composite scores, with three assessing the structural validity^28^, ^30^, ^36^ and one evaluating measurement invariance.^30^ Proficient structural validity and measurement invariance evidence for the Crystallized composite score support its scoring inference, confirming it adequately reflects key aspects of performance for older adult and ADRD populations. The Fluid composite score demonstrated developing evidence to support the scoring inference. Evidence related to the structural validity of the Fluid composite revealed two significant outcomes: (1) the two‐factor model demonstrated a good fit for individuals aged > 65, but a three‐factor solution was found for individuals aged < 65 years^30^; and (2) episodic memory and working memory demonstrated high missing rates for individuals with MCI/ADRD,^28^, ^30^, ^36^ with episodic memory less strongly associated with other Fluid cognition measures in this group.^30^ This missing data pattern aligns with Ho et al.’s^57^ findings and is significant given the rapid decline of Fluid abilities in early ADRD.^20^ Scoring inferences are heavily influenced by the specific design of test items (e.g., wording, detailed task specification), and most articles lacked discussion on trained examiners’ facilitation. Whether increased examiner assistance improves accessibility is unclear but warrants research. Many studies also grouped patients across the ADRD continuum without specifying symptom severity. Future studies should stratify these populations by cognitive abilities or provide more context on deficit severity.

Evidence supporting the generalization inference for the NIHTB‐CB was provided by five papers. Of the five studies reviewed, one provided evidence solely for internal consistency,^31^ three provided reliability statistics,^32^, ^34^, ^35^ and one provided statistics for both internal consistency and reliability^11^ of the Total, Crystallized, and/or Fluid composite scores. Evidence for the generalization inference of the Total, Fluid, and Crystallized composite scores was overwhelmingly rated as exemplary. Evidence for the internal consistency of the Fluid composite score was given a proficient rating for inconsistent results. In summary, the evidence indicates these synthesized composite scores accurately reflect performance across the test domains.

Support for the extrapolation inference of the Total, Fluid, and/or Crystallized composite was provided by seven papers assessing measurement error, criterion validity, and construct validity (i.e., convergent/discriminant validity, known group differences). Criterion validity evidence was exemplary in two papers that differed in the gold standard cognitive batteries used, as detailed in section 3.5.2.^11^, ^35^ Four studies^25^, ^26^, ^29^, ^33^ provided exemplary evidence of known group differences. Convergent/discriminant validity showed inconsistent results, with one study noting high correlations between Fluid and Crystallized measures for both the NIHTB‐CB and their corresponding gold standard tests in the low education (i.e., 10–12 years) group.^31^ Convergent/discriminant validity evidence from both studies^11^, ^31^ was rated proficient.

Only one study^35^ explored measurement error for the Fluid and Crystallized composite scores in older adult and ADRD populations, comparing them against specific gold standard cognitive measures (as outlined in section 3.5.2). Results indicated potentially “imprecise” results for Fluid and Crystallized composites compared to gold standards but noted no consistent bias. Preliminary analyses found the NIHTB‐CB may overestimate Fluid cognition for high‐scoring participants on its gold standard comparator and underestimate it for those with lower scores. Exploratory analyses also found the NIHTB Fluid composite tended to overestimate gold standard Fluid cognition comparators in younger participants and underestimate gold standard Fluid cognition in participants who identify as Black. These differences may reflect disparities in uncorrected scores that may be addressed using fully corrected scores. However, future studies should use an intersectional lens to fully understand, seek out, and study the compounded interactions of other factors that may contribute to these differences. Caution is warranted given this single study's small sample size (*N* = 61). Thus, measurement error evidence for Fluid and Crystallized composites was rated as developing. Future research should explore measurement error in older adults and ADRD and standardize gold standard measures. In summary, the evidence provides a solid foundation for supporting the extrapolation inference, with composites reflecting meaningful real‐life performance.

To date, no studies meeting our inclusion criteria have provided evidence to support the implication inference of the NIHTB‐CB composite scores. Even though these types of studies are more difficult to conduct,^16^, ^17^ this type of evidence is crucial for making informed decisions (e.g., making accurate clinical diagnoses, evaluating intervention efficacy, and guiding treatment planning) based on NIHTB‐CB performance in older adult and ADRD populations. Review findings suggest future research should focus efforts on three key areas. First, ensuring the Fluid composite score captures the most crucial elements of performance for older adult and ADRD populations (i.e., scoring inference evidence for the Fluid composite scores). This would involve studies rigorously examining the correspondence between Fluid composite scores and specific clinically relevant cognitive abilities, potentially using expert ratings or detailed cognitive task analyses. Second, characterizing the measurement error of the NIHTB‐CB composite score in diverse older populations and in individuals with various neurological disorders is vital. Understanding measurement error is crucial for interpreting individual score changes over time, determining statistically significant differences between groups, and enhancing the power of research aiming to detect subtle cognitive changes. Third, providing robust support for the implication inference of the NIHTB‐CB composite scores is essential. This necessitates future studies using longitudinal designs that correlate NIHTB‐CB scores with critical real‐world clinical outcomes (e.g., functional independence, quality of life, responsiveness to cognitive interventions, etc.), thereby demonstrating the direct clinical utility of the assessment.

Results of this systematic review highlight evidence for the psychometric robustness of the NIHTB‐CB in older adult and ADRD populations. While overall evidence was limited by minimal literature meeting IUA and COSMIN inclusion criteria, proficient to exemplary evidence was found for the Crystallized and Total composites. Measurement error for the Crystallized composite was an exception, likely due to small sample size. The Fluid composite also showed proficient to exemplary evidence in multiple properties, though more replication is needed to understand its structural validity, measurement invariance, and measurement error in these populations. The NIHTB‐CB offers a valuable middle ground for diagnosing MCI or dementia of the AD type, situated between brief cognitive screeners and lengthy neuropsychological evaluations. This comprehensive and efficient digital suite facilitates standardization and uses computer‐adaptive testing to minimize participant burden, making it advantageous across a wide range of settings. Further supporting its utility, a recent study by Ho et al.^57^ found the NIHTB‐CB to be a valid set of measures for characterizing and differentiating among groups with normal cognition, MCI, and dementia of the AD type. Moreover, these findings are consistent with other studies demonstrating the sensitivity of several NIHTB fluid measures compared to standard neuropsychological tests.^35^ The findings of this review substantiate and extend these results, advocating for the continued application and utility of the NIHTB‐CB in diverse older adult populations. To our knowledge, this is the first systematic review of the psychometric properties of the NIHTB‐CB composite scores in older adult populations who are most at risk of ADRD. Results suggest future research must continue to study the NIHTB‐CB in older adults at risk for ADRD and prioritize efforts to recruit participants from diverse racial/ethnic backgrounds to truly address ADRD as a growing public health crisis.

## CONFLICT OF INTEREST STATEMENT

The authors have no conflicts or competing interests to declare. Author disclosures are available in the supporting information.

## Supporting information



Supporting Information

Supporting Information
