# Supplementary material for: Psychometric properties of the NIH Toolbox Cognition Battery composites in older adults at risk for Alzheimer's disease and related dementias: A systematic review
Source: Alzheimers Dement. 2025 Oct 28;21(10):e70673. doi: 10.1002/alz.70673 (PMC12560014; doi:10.1002/alz.70673)
Supplement: Supplementary file 1 — Supporting Information [file ALZ-21-e70673-s002.docx]

***Key Concepts***

- NIH Toolbox Cognition Battery
- Validity
- Reliability
- Dementia
- Alzheimer's Disease
- Psychometrics
- Norms/Normative Data

***Variations of Key Concepts (Keywords and MeSH/Emtree Terms)***

- **NIH Toolbox Cognition Battery**
  - "National Institutes of Health Toolbox Cognition Battery" (Exact Phrase)
  - "NIH Toolbox Cognition Battery" (Exact Phrase)
  - "Toolbox Cognition Battery" (Exact Phrase)
  - "NIHTB-CB" (Acronym)
  - "national institutes of health (u.s.)"[MeSH Terms]
  - "national institutes of health"[All Fields]
  - "national institutes of health (u.s.)"[All Fields]
  - "national institutes of health u s"[MeSH Terms]
  - "nih"[All Fields]
  - "Toolbox"[All Fields] / "toolbox"[All Fields] / "toolboxes"[All Fields]
  - "Cognition"[MeSH Terms] / "cognition"[All Fields] / "cognitions"[All Fields] / "cognitive"[All Fields] / "cognitively"[All Fields] / "cognitives"[All Fields]
  - "Battery"[All Fields] / "batterie"[All Fields] / "batteries"[All Fields] / "battery s"[All Fields]
  - 'nih toolbox cognition battery'/exp (Embase controlled vocabulary)
- **Validity:**
  - "Validity"[All Fields]
  - "validity"[All Fields]
  - "validation study"[All Fields]
  - "validation study"[MeSH Terms] / 'validation study'/exp
  - "instrument"[All Fields] AND "validity"[All Fields]
  - 'validity'/exp (Embase controlled vocabulary)
  - 'validation study'/de (Embase descriptor)
- **Reliability:**
  - "Reliability"[All Fields]
  - "reliability"[All Fields]
  - "reproducibility"[All Fields] / "reproducibility of results"[MeSH Terms] / 'reproducibility'/exp
  - "Repeatability"[All Fields]
  - ("research design"[MeSH Terms] OR "research design"[All Fields] OR "test"[All Fields]) AND "reliability"[All Fields]
  - "instrument"[All Fields] AND "reliability"[All Fields]
- **Dementia:**
  - "Dementia"[MeSH Terms] / "dementia"[All Fields] / "dementias"[All Fields] / "dementia s"[All Fields]
  - 'dementia'/exp (Embase controlled vocabulary)
- **Alzheimer's Disease:**
  - "Alzheimer disease"[MeSH Terms]
  - "alzheimer disease"[All Fields]
  - "alzheimer's disease"[All Fields]
  - "alzheimers"[All Fields] / "alzheimers s"[All Fields]
  - 'alzheimer disease'/exp (Embase controlled vocabulary)
- **Psychometrics:**
  - "Psychometrics"[MeSH Terms] / "psychometrics"[All Fields]
  - 'psychometry'/exp (Embase controlled vocabulary)
- **Norms/Normative Data:**
  - "norm"[All Fields]
  - "statistics and numerical data"[MeSH Subheading, specifically for NIH, Neuropsychological Tests, Reproducibility of Results]

***Filters***

- **Publication Type/Status:**
  - "author manuscript"[Filter] (PubMed specific)
  - "Peer reviewed"
- **Age Groups:**
  - "Aged (65 Yrs & Older)"
  - "Middle Age (40-64 Yrs)" / "Middle Aged: 45-64 years"
  - "Very Old (85 Yrs & Older)" / "Aged, 80 and over" / "80 and over: 80+ years"
  - "middleagedaged"[Filter] (PubMed specific, covers 45+?)
  - "[aged]/lim" (Embase specific)
  - "[middle aged]/lim" (Embase specific)
  - "SubjectAge: - aged: 65+ years" (CINHAL specific)
- **Language:**
  - "English language" / "English"
- **Publication Date:**
  - "2013/01/01"[PubDate] : "3000/12/31"[PubDate] (PubMed specific, covers 2013 onwards)
  - "2013:2021"[pdat] (PubMed specific, covers 2013-2024)
- **Population Type:**
  - "Humans"

**Databases Used**

- MEDLINE
- PubMed (note: PubMed includes MEDLINE, so there is an overlap in results)
- CINAHL
- PsycINFO
- Embase

**Database-Specific Terms**

| **Database** | **Search Query (Exact Syntax Used)** | **Filters Applied** |
| --- | --- | --- |
| **MEDLINE** | (((((National Institutes of Health Toolbox Cognition Battery) OR NIH Toolbox Cognition Battery) OR Toolbox Cognition Battery) AND Validity) AND Dementia)  (("national institutes of health (u.s.)"[MeSH Terms] OR ("national"[All Fields] AND "institutes"[All Fields] AND "health"[All Fields] AND "(u.s.)"[All Fields]) OR "national institutes of health (u.s.)"[All Fields] OR ("national"[All Fields] AND "institutes"[All Fields] AND "health"[All Fields]) OR "national institutes of health"[All Fields]) AND Toolbox[All Fields] AND ("cognition"[MeSH Terms] OR "cognition"[All Fields]) AND Battery[All Fields]) AND Validity[All Fields]) AND ("dementia"[MeSH Terms] OR "dementia"[All Fields]) AND "author manuscript"[Filter]  ("national institutes of health (u.s.)"[MeSH Terms] OR ("national"[All Fields] AND "institutes"[All Fields] AND "health"[All Fields] AND "(u.s.)"[All Fields]) OR "national institutes of health (u.s.)"[All Fields] OR ("national"[All Fields] AND "institutes"[All Fields] AND "health"[All Fields]) OR "national institutes of health"[All Fields]) AND Toolbox[All Fields] AND ("cognition"[MeSH Terms] OR "cognition"[All Fields]) AND Battery[All Fields]) AND Validity[All Fields] AND ("alzheimer disease"[MeSH Terms] OR ("alzheimer"[All Fields] AND "disease"[All Fields]) OR "alzheimer disease"[All Fields] OR ("alzheimer's"[All Fields] AND "disease"[All Fields]) OR "alzheimer's disease"[All Fields]) AND "author manuscript"[Filter] | Authored manuscripts |
| **PubMed** | ((((((((((((("national institutes of health (u.s.)"[MeSH Terms] OR ("national"[All Fields] AND "institutes"[All Fields] AND "health"[All Fields] AND "(u.s.)"[All Fields]) OR "national institutes of health (u.s.)"[All Fields] OR "nih"[All Fields]) AND Toolbox[All Fields] AND ("cognition"[MeSH Terms] OR "cognition"[All Fields]) AND Battery[All Fields]) OR NIHTB-CB[All Fields]) AND validity[All Fields]) OR (instrument[All Fields] AND validity[All Fields])) AND "reliability"[All Fields]) OR (("research design"[MeSH Terms] OR ("research"[All Fields] AND "design"[All Fields]) OR "research design"[All Fields] OR "test"[All Fields]) AND "reliability"[All Fields])) OR (instrument[All Fields] AND "reliability"[All Fields])) AND reproducibility[All Fields]) OR Repeatability[All Fields]) AND ("dementia"[MeSH Terms] OR "dementia"[All Fields])) OR ("alzheimer disease"[MeSH Terms] OR ("alzheimer"[All Fields] AND "disease"[All Fields]) OR "alzheimer disease"[All Fields] OR "alzheimers"[All Fields])) AND "cognitive"[All Fields]) AND ("psychometrics"[MeSH Terms] OR "psychometrics"[All Fields]) AND "author manuscript"[Filter] AND ("author manuscript"[Filter] AND ("2013/01/01"[PubDate] : "3000/12/31"[PubDate]))  ((((((((((((("national institutes of health (u.s.)"[MeSH Terms] OR ("national"[All Fields] AND "institutes"[All Fields] AND "health"[All Fields] AND "(u.s.)"[All Fields]) OR "national institutes of health (u.s.)"[All Fields] OR "nih"[All Fields]) AND Toolbox[All Fields] AND ("cognition"[MeSH Terms] OR "cognition"[All Fields]) AND Battery[All Fields]) OR NIHTB-CB[All Fields]) AND validity[All Fields]) OR (instrument[All Fields] AND validity[All Fields])) AND "reliability"[All Fields]) OR (("research design"[MeSH Terms] OR ("research"[All Fields] AND "design"[All Fields]) OR "research design"[All Fields] OR "test"[All Fields]) AND "reliability"[All Fields])) OR (instrument[All Fields] AND "reliability"[All Fields])) AND reproducibility[All Fields]) OR Repeatability[All Fields]) AND ("dementia"[MeSH Terms] OR "dementia"[All Fields])) OR ("alzheimer disease"[MeSH Terms] OR ("alzheimer"[All Fields] AND "disease"[All Fields]) OR "alzheimer disease"[All Fields] OR "alzheimers"[All Fields])) AND "cognitive"[All Fields]) AND ("psychometrics"[MeSH Terms] OR "psychometrics"[All Fields]) AND "norm"[All Fields] AND "author manuscript"[Filter] AND ("author manuscript"[Filter] AND ("2013/01/01"[PubDate] : "3000/12/31"[PubDate]))  ( (((("national institutes of health u s /statistics and numerical data"[MeSH Terms] AND "neuropsychological tests/statistics and numerical data"[MeSH Terms]) OR "reproducibility of results/statistics and numerical data"[MeSH Terms] OR "Psychometrics"[MeSH Terms]) AND ("dementia"[MeSH Terms] OR "dementia"[All Fields] OR "dementias"[All Fields] OR "dementia s"[All Fields])) OR ("alzheime s"[All Fields] OR "alzheimer disease"[MeSH Terms] OR ("alzheimer"[All Fields] AND "disease"[All Fields]) OR "alzheimer disease"[All Fields] OR "alzheimer"[All Fields] OR "alzheimers"[All Fields] OR "alzheimer s"[All Fields] OR "alzheimers s"[All Fields])) AND (("toolbox"[All Fields] OR "toolboxes"[All Fields]) AND ("cognition"[MeSH Terms] OR "cognition"[All Fields] OR "cognitions"[All Fields] OR "cognitive"[All Fields] OR "cognitively"[All Fields] OR "cognitives"[All Fields]) AND ("batterie"[All Fields] OR "batteries"[All Fields] OR "battery"[All Fields] OR "battery s"[All Fields]))) OR (("national institutes of health u s"[MeSH Terms] OR ("national"[All Fields] AND "institutes"[All Fields] AND "health"[All Fields] AND "us"[All Fields]) OR "national institutes of health u s"[All Fields] OR "nih"[All Fields]) AND ("toolbox"[All Fields] OR "toolboxes"[All Fields]) AND ("cognition"[MeSH Terms] OR "cognition"[All Fields] OR "cognitions"[All Fields] OR "cognitive"[All Fields] OR "cognitively"[All Fields] OR "cognitives"[All Fields]) AND ("batterie"[All Fields] OR "batteries"[All Fields] OR "battery"[All Fields] OR "battery s"[All Fields])) OR (("national institutes of health u s"[MeSH Terms] OR ("national"[All Fields] AND "institutes"[All Fields] AND "health"[All Fields] AND "us"[All Fields]) OR "national institutes of health u s"[All Fields] OR ("national"[All Fields] AND "institutes"[All Fields] AND "health"[All Fields]) OR "national institutes of health"[All Fields]) AND ("toolbox"[All Fields] OR "toolboxes"[All Fields]) AND ("cognition"[MeSH Terms] OR "cognition"[All Fields] OR "cognitions"[All Fields] OR "cognitive"[All Fields] OR "cognitively"[All Fields] OR "cognitives"[All Fields]) AND ("batterie"[All Fields] OR "batteries"[All Fields] OR "battery"[All Fields] OR "battery s"[All Fields])) OR "NIHTB-CB"[All Fields]) AND ((fft[Filter]) AND (2013:2021[pdat]))  ((((National Institutes of Health Toolbox Cognition Battery) OR NIH Toolbox Cognition Battery) OR Toolbox Cognition Battery) AND Validity) AND Dementia)  NIHTB-CB or NIH Toolbox cognition battery AND ("Cognition"[MeSH]) AND "Neuropsychological Tests"[MeSH]) AND "Cognitive Dysfunction/diagnosis"[MAJR]) AND "Cognition"[MeSH  ("national institutes of health u s"[MeSH Terms] OR ("national"[All Fields] AND "institutes"[All Fields] AND "health"[All Fields] AND "u s"[All Fields]) OR "national institutes of health u s"[All Fields] OR "nih"[All Fields]) AND ("toolbox"[All Fields] OR "toolboxes"[All Fields]) AND ("cognition"[MeSH Terms] OR "cognition"[All Fields] OR "cognitions"[All Fields] OR "cognitive"[All Fields] OR "cognitively"[All Fields] OR "cognitives"[All Fields]) AND ("batterie"[All Fields] OR "batteries"[All Fields] OR "battery"[All Fields] OR "battery s"[All Fields])) AND ((fft[Filter]) AND (middleagedaged[Filter]) AND (2021:2023[pdat]))  (( (((("national institutes of health u s /statistics and numerical data"[MeSH Terms] AND "neuropsychological tests/statistics and numerical data"[MeSH Terms]) OR "reproducibility of results/statistics and numerical data"[MeSH Terms] OR "Psychometrics"[MeSH Terms]) AND ("dementia"[MeSH Terms] OR "dementia"[All Fields] OR "dementias"[All Fields] OR "dementia s"[All Fields])) OR ("alzheime s"[All Fields] OR "alzheimer disease"[MeSH Terms] OR ("alzheimer"[All Fields] AND "disease"[All Fields]) OR "alzheimer disease"[All Fields] OR "alzheimer"[All Fields] OR "alzheimers"[All Fields] OR "alzheimer s"[All Fields] OR "alzheimers s"[All Fields])) AND (("toolbox"[All Fields] OR "toolboxes"[All Fields]) AND ("cognition"[MeSH Terms] OR "cognition"[All Fields] OR "cognitions"[All Fields] OR "cognitive"[All Fields] OR "cognitively"[All Fields] OR "cognitives"[All Fields]) AND ("batterie"[All Fields] OR "batteries"[All Fields] OR "battery"[All Fields] OR "battery s"[All Fields]))) OR (("national institutes of health u s"[MeSH Terms] OR ("national"[All Fields] AND "institutes"[All Fields] AND "health"[All Fields] AND "u s"[All Fields]) OR "national institutes of health u s"[All Fields] OR "nih"[All Fields]) AND ("toolbox"[All Fields] OR "toolboxes"[All Fields]) AND ("cognition"[MeSH Terms] OR "cognition"[All Fields] OR "cognitions"[All Fields] OR "cognitive"[All Fields] OR "cognitively"[All Fields] OR "cognitives"[All Fields]) AND ("batterie"[All Fields] OR "batteries"[All Fields] OR "battery"[All Fields] OR "battery s"[All Fields])) OR (("national institutes of health u s"[MeSH Terms] OR ("national"[All Fields] AND "institutes"[All Fields] AND "health"[All Fields] AND "u s"[All Fields]) OR "national institutes of health u s"[All Fields] OR ("national"[All Fields] AND "institutes"[All Fields] AND "health"[All Fields]) OR "national institutes of health"[All Fields]) AND ("toolbox"[All Fields] OR "toolboxes"[All Fields]) AND ("cognition"[MeSH Terms] OR "cognition"[All Fields] OR "cognitions"[All Fields] OR "cognitive"[All Fields] OR "cognitively"[All Fields] OR "cognitives"[All Fields]) AND ("batterie"[All Fields] OR "batteries"[All Fields] OR "battery"[All Fields] OR "battery s"[All Fields])) OR "NIHTB-CB"[All Fields]) AND ((fft[Filter]) AND (2013:2021[pdat]))  NIHTB-CB or NIH Toolbox cognition battery AND ("Cognition"[MeSH]) AND "Neuropsychological Tests"[MeSH]) AND "Cognitive Dysfunction/diagnosis"[MAJR]) AND "Cognition"[MeSH  (("national institutes of health u s"[MeSH Terms] OR ("national"[All Fields] AND "institutes"[All Fields] AND "health"[All Fields] AND "u s"[All Fields]) OR "national institutes of health u s"[All Fields] OR "nih"[All Fields]) AND ("toolbox"[All Fields] OR "toolboxes"[All Fields]) AND ("cognition"[MeSH Terms] OR "cognition"[All Fields] OR "cognitions"[All Fields] OR "cognitive"[All Fields] OR "cognitively"[All Fields] OR "cognitives"[All Fields]) AND ("batterie"[All Fields] OR "batteries"[All Fields] OR "battery"[All Fields] OR "battery s"[All Fields])) AND ((fft[Filter]) AND (middleagedaged[Filter]) AND (2023:2024[pdat]))  (("national institutes of health (u.s.)"[MeSH Terms] OR ("national"[All Fields] AND "institutes"[All Fields] AND "health"[All Fields] AND "(u.s.)"[All Fields]) OR "national institutes of health (u.s.)"[All Fields] OR ("national"[All Fields] AND "institutes"[All Fields] AND "health"[All Fields]) OR "national institutes of health"[All Fields]) AND Toolbox[All Fields] AND ("cognition"[MeSH Terms] OR "cognition"[All Fields]) AND Battery[All Fields]) AND Validity[All Fields] AND ("alzheimer disease"[MeSH Terms] OR ("alzheimer"[All Fields] AND "disease"[All Fields]) OR "alzheimer disease"[All Fields] OR ("alzheimer's"[All Fields] AND "disease"[All Fields]) OR "alzheimer's disease"[All Fields]) AND "author manuscript"[Filter] | Authored manuscripts  Peer reviewed  Age group: Aged (65 Yrs & Older), Middle Age (40-64 Yrs), Very Old (85 Yrs & Older), All adult  Population: Human  English Language |
| **CINAHL** | National Institutes of Health Toolbox Cognition Battery OR NIH Toolbox Cognition Battery OR Toolbox Cognition Battery AND Validity AND Alzheimer’s Disease  National Institutes of Health Toolbox Cognition Battery OR NIH Toolbox Cognition Battery OR Toolbox Cognition Battery AND Validity AND Dementia  NIH Toolbox Cognition Battery OR Toolbox Cognition Battery AND Reliability AND dementia or alzheimers  NIH Toolbox Cognition Battery OR Toolbox Cognition Battery AND validity and reliability AND dementia or alzheimers  NIH Toolbox Cognition Battery) OR (Toolbox Cognition Battery ) OR (National Institutes of Health Toolbox Cognition Battery) AND Validity AND Dementia  National Institutes of Health Toolbox Cognition Battery OR NIH Toolbox Cognition Battery OR Toolbox Cognition Battery AND Validity AND Alzheimer’s Disease  National Institutes of Health Toolbox Cognition Battery OR NIH Toolbox Cognition Battery OR Toolbox Cognition Battery AND Validity AND Alzheimer’s Disease | Subject Age: - aged: 65+ years  English Language  Age Groups: Middle Aged: 45-64 years, Aged: 65+ years, Aged, 80 and over  Peer reviewed |
| **PsycINFO** | (NIH Toolbox Cognition Battery ) OR (Toolbox Cognition Battery ) OR (National Institutes of Health Toolbox Cognition Battery ) AND Validity AND Dementia  Toolbox Cognition Battery) OR (National Institutes of Health Toolbox Cognition Battery) AND Reliability AND Dementia AND alzheimers’s Disease  Toolbox Cognition Battery) OR (National Institutes of Health Toolbox Cognition Battery) AND validity AND alzheimers  NIH Toolbox Cognition Battery ) OR (Toolbox Cognition Battery ) OR (National Institutes of Health Toolbox Cognition Battery ) AND Validity AND Dementia  Toolbox Cognition Battery) OR (National Institutes of Health Toolbox Cognition Battery) AND Reliability AND Dementia AND alzheimers’s Disease | Peer reviewed  Age group: Aged (65 Yrs & Older), Middle Age (40-64 Yrs), Very Old (85 Yrs & Older) all adult Population, Human |
| **EMBASE** | ('nih toolbox cognition battery'/exp OR 'nih toolbox cognition battery' OR (nih AND toolbox AND ('cognition'/exp OR cognition) AND ('battery'/exp OR battery))) AND ('validation study'/exp OR 'validation study') AND reliability AND dementia  'nih toolbox cognition battery'/exp OR 'nih toolbox cognition battery' OR (nih AND toolbox AND ('cognition'/exp OR cognition) AND ('battery'/exp OR battery))) AND ('validation study'/exp OR 'validation study') AND reliability AND dementia  'nih toolbox cognition battery'/exp OR 'nih toolbox cognition battery' OR (nih AND toolbox AND ('cognition'/exp OR cognition) AND ('battery'/exp OR battery))) AND ('validity'/exp OR validity) AND reliability AND [aged]/lim OR [middle aged]/lim)  'nih toolbox cognition battery'/exp OR 'nih toolbox cognition battery' OR (nih AND toolbox AND ('cognition'/exp OR cognition) AND ('battery'/exp OR battery))) AND ('validity'/exp OR validity) AND reliability  ('nih toolbox cognition battery'/exp OR 'nih toolbox cognition battery' OR (nih AND toolbox AND ('cognition'/exp OR cognition) AND ('battery'/exp OR battery))) AND ('validation study'/exp OR 'validation study') AND reliability AND dementia  'nih toolbox cognition battery'/exp OR 'nih toolbox cognition battery' OR (nih AND toolbox AND ('cognition'/exp OR cognition) AND ('battery'/exp OR battery))) AND ('validity'/exp OR validity) AND reliability AND psychometry  ('nih toolbox cognition battery'/exp OR 'nih toolbox cognition battery' OR (nih AND toolbox AND ('cognition'/exp OR cognition) AND ('battery'/exp OR battery))) AND ('validity'/exp OR validity) AND reliability  ('nih toolbox cognition battery'/exp OR 'nih toolbox cognition battery' OR (nih AND toolbox AND ('cognition'/exp OR cognition) AND ('battery'/exp OR battery))) AND ('validity'/exp OR validity) AND ‘validation study’/de  ‘nihtb cb' AND ('reproducibility'/exp OR reproducibility)  nihtb cb' AND ('alzheimer disease'/exp OR 'alzheimer disease')  nihtb cb' AND ('alzheimer disease'/exp OR 'alzheimer disease') AND ‘norm’  ((('nihtb cb' OR 'nih toolbox cognition battery'/exp OR 'nih toolbox cognition battery') AND ('validation study'/exp OR 'validation study') OR 'reliability'/exp OR reliability) AND ('dementia'/exp OR dementia) OR 'alzheimer disease'/exp OR 'alzheimer disease') AND ('psychometry'/exp OR psychometry) AND ('repeatability'/exp OR repeatability) | 45-64 years old,  65+ years |
